# Time- and concentration-dependent cytotoxicity of antibiotics used in endodontic therapy

**DOI:** 10.1590/S1678-77572010000300011

**Published:** 2010

**Authors:** Marina Beloti FERREIRA, Suely MYIAGI, Carlos Goes NOGALES, Marcia Sampaio CAMPOS, José Luiz LAGE-MARQUES

**Affiliations:** 1 DDS, MsC, Department of Restorative Dentistry, Discipline of Endodontics, Dental School of University of São Paulo, São Paulo, Brazil.; 2 DDS, PhD, Department of Restorative Dentistry, Discipline Of Endodontics, Dental School of University of São Paulo, São Paulo, Brazil.; 4 DDS, PhD, Department of Pathology, Dental School of University of São Paulo, São Paulo, Brazil.; 5 Associate Professor of Restorative and Endodontics, Department of the Dental School of University of São Paulo.

**Keywords:** Cytotoxicity, Ciprofloxacin, Clyndamicin, Metronidazole

## Abstract

**Objective:**

New drugs have to be assessed in endodontic therapy due to the presence of microorganisms resistant to therapeutic procedures. Thus, this study evaluated the time- and concentration-dependent cytotoxicity of different antibiotics used in endodontic therapy.

**Material and Methods:**

Human gingival fibroblasts were treated and divided into the following experimental groups: Group I - control; Group II - ciprofloxacin hydrochloride; Group III - clyndamicin hydrochloride; and Group IV - metronidazole. Each drug was used at concentrations of 5, 50, 150, and 300 mg/L for 24, 48, 72, and 96 h. Cytotoxicity was evaluated by the MTT assay [3-(4,5-dimethylthiazol-2-yl)-2,5-diphenyltetrazolium bromide] and spectrophotometric reading of ELISA plates. The results were analyzed by BioEstat 4.0 software using Kruskal-Wallis and Dunn’s tests at a significance level of 5%. Cell viability was assessed for the different concentrations and times.

**RESULTS:**

All drugs presented dose-dependent cytotoxicity. Concentrations of 5 and 50 mg/L produced viable fibroblasts at all experimental times in all groups.

**Conclusions:**

Cell viability at 24 h was greater than in the other experimental times. Comparison between the same concentrations of antibiotics at different times showed that metronidazole presented the highest cell viability at 72 and 96 h compared to the other antibiotics, whereas clyndamicin hydrochloride showed higher cell viability at 72 h than ciprofloxacin hydrochloride.

## INTRODUCTION

Successful endodontic treatment involves the removal of the etiological agent, which most of the times is a microorganism. Although chemomechanical preparation aims at eliminating microorganisms from the root canal system, this procedure may not be sufficient to eliminate the focus of infection because some pathogens remain viable in the main canal and in dentinal tubules, needing dentin desmineralization and intracanal dressing.

In other clinical situations, not even the association of chemomechanical preparation and drug therapy is effective in eliminating endodontic infections because of the presence of microorganisms resistant to drugs and chemical agents, and the formation of biofilms in the periapical region. These situations require alternative interventions, such as the combination of antibiotics to achieve adequate concentrations in the dentinal tubules, so that they can act in areas that are not reached by the endodontic instruments and irrigating solutions, and kill resistant and facultative anaerobic microorganisms.

Among the drugs commonly used for endodontic infections, ciprofloxacin is indicated due to its efficient action against oral anaerobes, gram-positive aerobic microorganisms (*Staphylococcus aureus, S. epidermidis, Sptreptococcus spp*) and gram-negative enterobacteria (*Escherichia coli, Enterobacter spp* and *Pseudomonas*), which show MIC_90_ between 0.015 and 2 µg/mL. All streptococcal species are sensitive to concentrations between 1.0 and 8.0 µg/mL; *S. aureus* and *S. epidermidis* are sensitive to concentrations between 0.25 and 1.0 µg/mL^10^. Metronidazole has a unique spectrum of activity, covering strict anaerobic Gram-positive and Gram-negative bacteria, and protozoa^3^. Its bactericidal action involves breaking bacterial DNA and inhibiting nucleic acid synthesis, and affects almost all gram-negative anaerobic bacilli^5^. Clyndamicin acts on resistant root canal microbiota, gram-positive aerobic bacilli, such as *S. aureus, S. epidermidis* and *Pneumococci*, as well as on gram-positive and gram-negative bacteria^10^.

In addition to the antimicrobial action, the cytotoxicity of antibiotics used in endodontic therapy should be determined, as it may provide a scientific basis for professionals making a decision on the most biocompatible drugs to be used. The aim of this study was to assess the cytotoxicity of ciprofloxacin hydrochloride, clyndamicin hydrochloride and metronidazole on human gingival fibroblast cultures.

## MATERIAL AND METHOD

The human gingival fibroblasts (FMM1) used in this study were donated by the Basic Research Laboratory of the Dental School of the University of São Paulo, Brazil. The present study was approved by the Research ethics Committee of the University of São Paulo (Protocol number 02/05).

**Figure 1:**
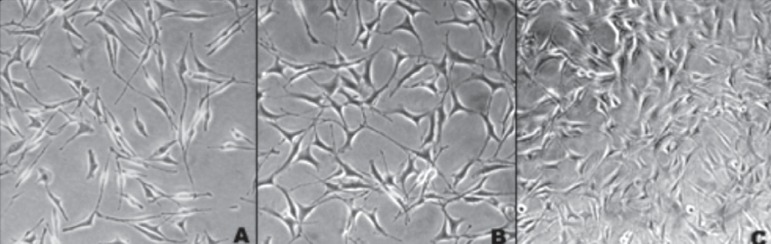
Monitoring fibroblast growth. (A) and (B) fibroblast increasing; (C) cells in subconfluence

Cells were thawed in water at 37°C for 30 s and transferred toa 65cm^2^ cell culturebottlecontaining 15mL of culture broth. Cells were kept at 37°C in moist environment with 95% air and 5% CO_2_. Cell growth was assessed every 24 h with an inverted phase microscope, until cells were confluent (Figure 1). Broth was changed every other day in order to maintain cell viability. Cells were subcultured to the sixth passage, when a standard number of cells were obtained for the assay.

In order to determine the number of cells in the original flasks, cells were submitted to trypsin treatment and transferred to a test tube that was centrifuged at 300 rmp for 5 min at room temperature. Cells were counted in a Neubauer chamber^5^ , and Dulbecco’s Modified Eagle Medium (DMeM) was added to the original bottles in a sufficient amount to produce 10^3^ cells in each 200 µL-well of the culture plate^4^.

One 96-well plate was used for each of the following experimental times: 24, 48, 72, and 96 h. The wells were filled with 200 µL culture broth with 10^3^ cells/well, and the plates were kept in an incubator with 5% CO_2_ atmosphere at 37°C for 24 h for cell adherence.

The concentration of the drugs used in this assay followed the protocol proposed by Gürbay, et al.^7^ (2007). The following groups were formed: Group I: Control (cells in culture broth); Group II: ciprofloxacin hydrochloride (300, 150, 50 and 5 mg/L); Group III: Clyndamicin hydrochloride (300, 150, 50 and 5 mg/L); Group IV: Metronidazole gel 10% (300, 150, 50 and 5 mg/L). The drugs were prepared at the Basic Research Laboratory of the Dental School of the University of São Paulo, Brazil. each drug was diluted in distilled water and added to the culture broth (DMeM). experimental times of 24, 48, 72 and 96 h were used in all groups.

After 24 h of plating the cultures, broth was carefully aspirated in order not to break the monolayer. After that, 200 µL of each concentration of the tested drugs were added to the different plates. The control group was treated with 200 µL of culture broth. After 48 h, the culture medium of the plates incubated for 72 and 96 h was changed: the control group received fresh broth and the other plates, new dilutions of the antibiotics. After confirming the results, the assay was repeated other two times, totalizing three repetitions.

The mitochondrial activity of the fibroblasts was assessed by the MTT assay [3-(4,5-dimethylthiazol2-yl)-2,5-diphenyltetrazolium bromide] at the end of each experimental period. The contents of each well were gently stirred with a multichannel pipettor and submitted to absorbance reading at 560 nm in an eLISA spectrophotometer. Absorbance results were analyzed, converted in cell viability percentages and compared in the statistical analysis. The level of significance was set at 5%.

## RESULTS

**Figure 2:**
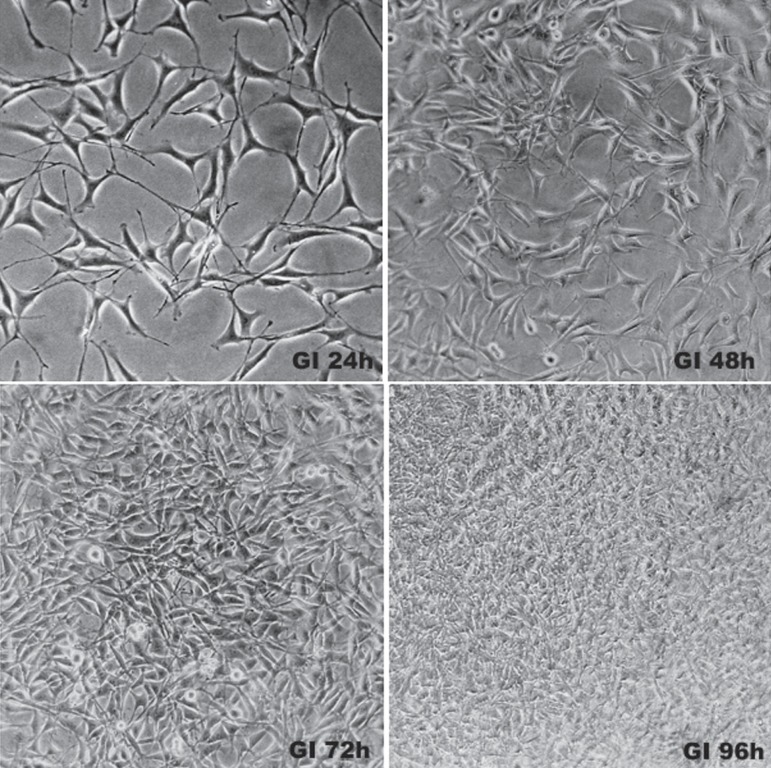
Photomicrographs of Group I (Control)

**Figure 3:**
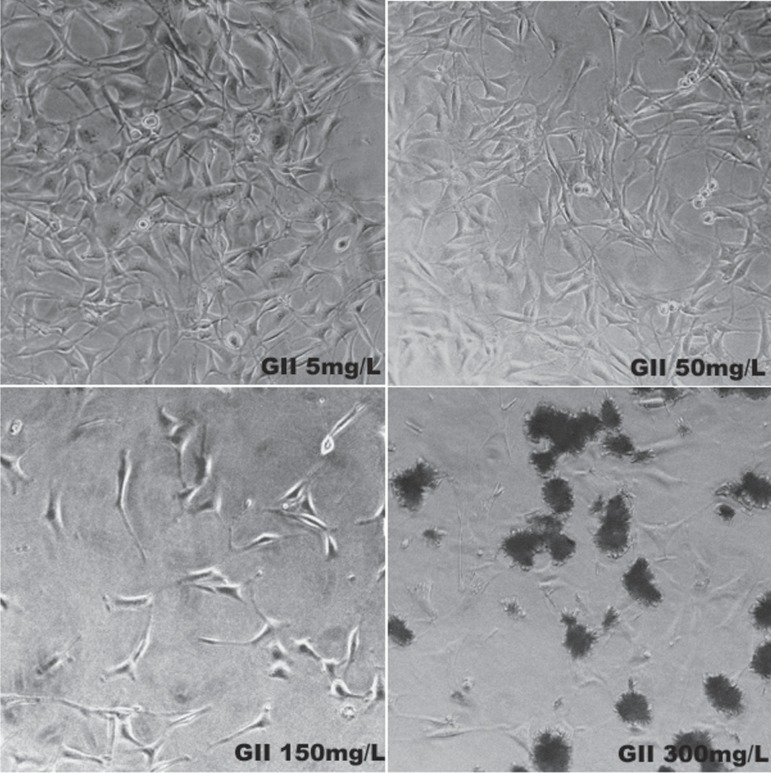
Photomicrographs of Group II (Ciprofloxacin) at 72 h

**Figure 4:**
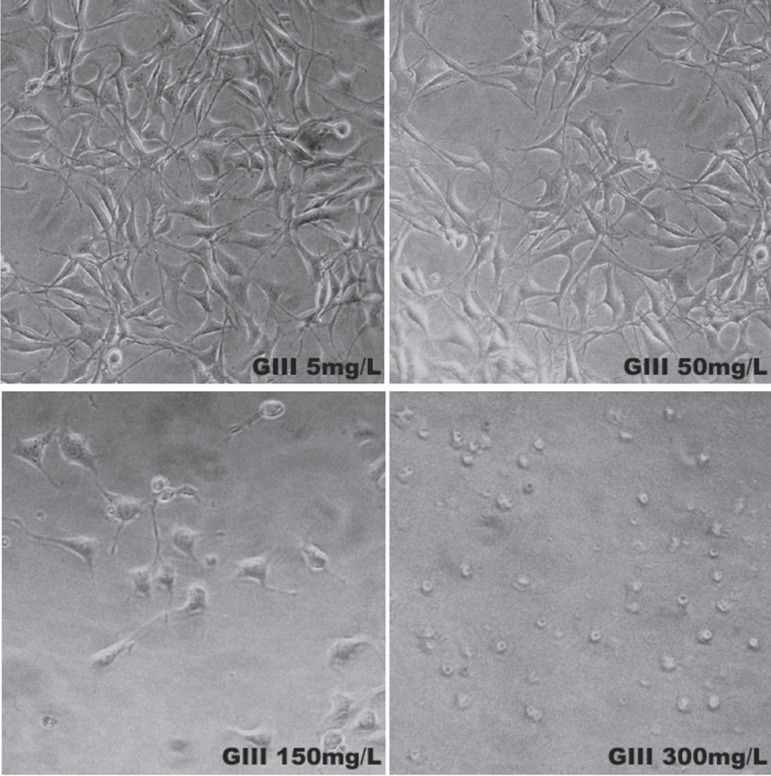
Photomicrographs of Group III (Clyndamicin) at 72 h

**Figure 5:**
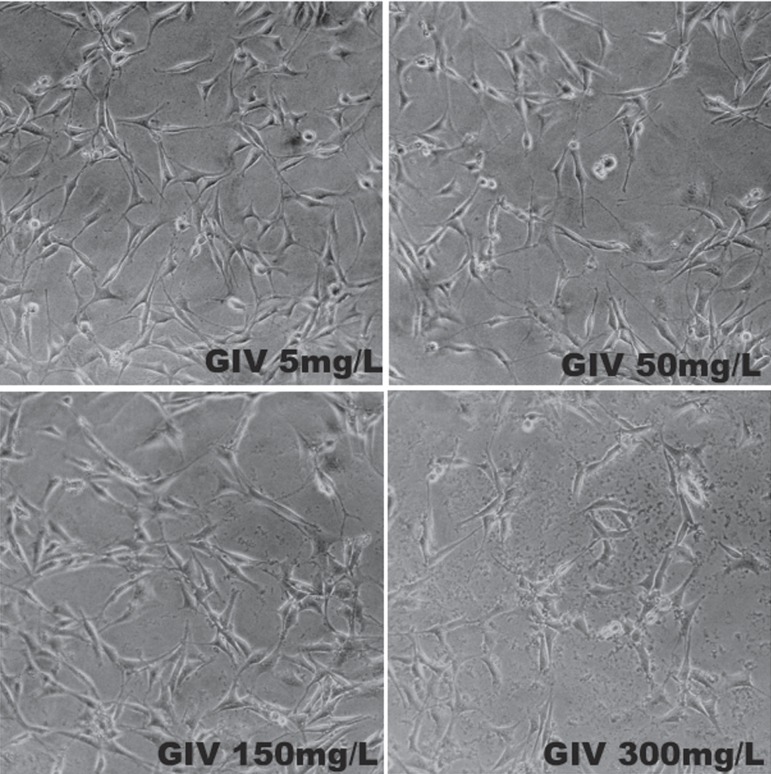
Photomicrographs of Group IV (Metronidazole) at 72 h

Data on mitochondrial activity obtained from the optical density of cell culture plates of the experimental groups were transformed in percentages in relation to the control group, considered to be 100%. These values are shown in Table 1 and illustrated in Figures 2 to 5, and are related to each group of standard fibroblast culture treated by different antibiotic concentrations for different experimental times.

**Table 1:** Mean cell viability (%) of fibroblasts according to the tested antibiotics, concentrations and experimental times

	**24 h**			**48h**			**72h**			**96h**		
	**CP**	**CL**	**M**	**CP**	**CL**	**M**	**CP**	**CL**	**M**	**CP**	**CL**	**M**
												
5 mg/L	78.61	70.06	71.01	55.84	66.16	68.15	83.80	77.32	71.34	69.88	81.68	73.95
50 mg/L	63.31	65.37	68.0	35.32	58.96	61.33	51.29	72.09	62.97	53.07	71.31	67.68
150 mg/L	37.56	45.35	62.75	26.87	36.16	56.97	33.39	42.74	56.33	29.31	30.50	62.90
300 mg/L	27.22	15.71	53.38	9.36	5.86	55.05	17.10	13.83	53.25	10.79	0.83	57.66

CP: Ciprofloxacin; CL: clyndamicin; M: metronidazole

Table 1 shows that at 24 h, 5 and 50 mg/L of ciprofloxacin produced at least 60% cell viability, decreasing in the next experimental period and increasing until 96 h. Concentrations of 150 and 300 mg/L produced the smallest number of viable cells at all experimental times. The Kruskal-Wallis test was used with the Dunn’s test because of the non-normal distribution of the number of viable cells. Significance level set at 5% for the different interactions between each antimicrobial agent and their different concentrations and experimental times.

Concentrations of 5 and 50 mg/L of clyndamicin produced about 60% viable cells at 24 and 48 h, and over 70% at the last two experimental times. Concentrations of 150 and 300 mg/L led to less than 50 and 20% of viable cells at 24 and 48 h and a decrease in the number of cells after 96 h.

Figure 2 shows normal fibroblasts in the control group. At 24 h, cells were fusiform with central nucleuses and typical cytoplasmic extensions, which have an important role in cell contact. At 48 h, there were more viable cells, occupying about 70% of the wells, representing the subconfluence state. At 96 h, cells were confluent and overlapping.

Figure 3 shows the ciprofloxacin-treated group. Representative images obtained for the concentrations of 5 and 50 mg/L showed that fibroblasts were fusiform with central nucleuses and typical cytoplasmic extensions. For the concentration of 150 mg/L, the smallest number of cells was observed with the greatest spacing between them. For the concentration of 300 mg/L, there were particles among the few existing cells suggesting the drug precipitated (Figure 3A).

Figure 4 shows the clyndamicin-treated group. Fibroblasts treated with 5 and 50 mg/L were fusiform with central nucleuses and typical cytoplasmic extensions. For the concentration of 150 mg/L, fewer, unattached, round cells with minimal cytoplasmic extensions were observed. Treatment with 300 mg/L produced the smallest number of viable cells, which were adherent, but had no defined shape.

Figure 5 presents the metronidazole-treated group. Cells were fusiform and slightly round when compared with the control group. For the concentrations of 150 and 300 mg/L, a large number of fibroblasts were seen in a disorganized arrangement, with a tendency to form clumps. Precipitated drug was observed in the bottom of the bottle.

## DISCUSSION

The methodology applied in this study was based on a previous study^6^ , which assessed biological effects of the ciprofloxacin on cell cultures. Cells selected for the assay – sixth-passage human gingival fibroblasts – were chosen due the ease of handling and metabolic potential similar to that of cells in the periapical region. It also is important to explain that the consumption of the nutrient broth by the cells is also responsible for their decreased viability. DMEM broth supplemented with 10% bovine fetal serum was chosen because it reproduces the ideal conditions for the *in vitro* maintenance of these cells.

The technique proposed to assess ciprofloxacin, clyndamicin and metronidazole cytotoxicity measured cell viability using the MTT assay. The efficacy of this method has been extensively demonstrated^2,6,7,11^.The results presented are related to the effects of three different antimicrobial compounds (ciprofloxacin, clyndamicin and metronidazole) at four different concentrations (5, 50, 150 and 300 mg/L) at four different times (24, 48, 72 and 96 h) on cells in culture.

Statistical interaction of ciprofloxacin concentrations showed significant differences between the following concentrations: 5x150 mg/L, 5x300 mg/L and 5x300 mg/L at 24 h; 5x300 mg/L at 48 h; 5x300 mg/L at 72 h; 5x150 mg/L, 5x300 mg/L and 50x300 mg/L at 96 h. According to these data and mean cell viability, the greatest concentrations produced the smallest number of viable cells compared to the control group. These results were similar to those of previous studies^6-7^ ;, which showed the cytotoxicity of ciprofloxacin at concentrations above 50 mg/L.

Statistical interaction of clyndamicin concentrations showed significant differences between the following concentrations: 5x300 mg/L, at 24 h; 5x300 mg/L at 48 h; 5x150 mg/L, 5x300 mg/L and 50x300 mg/L at 72 h; and finally 5x150 mg/L, 5x300 mg/L, 50x150 mg/L and 50x300 mg/L at 96 h. These results confirm those of Wijsman, et al.^11^ (2005) about the dose-dependent toxicity of clyndamicin.

Considering the antimicrobial action of these drugs, the findings of this study are in agreement with those of LeCorn, et al.^9^ (2007), who evaluated the susceptibility of several *Actinomyces* species to clyndamicin. Minimal inhibitory concentration of this antibiotic was 1 µg/mL.

All concentrations of metronidazole led to at least 50% viable cells at all concentrations at all experimental times. A concentration of 5 mg/L resulted in cell viability of 73% after 96 h.

Statistical interaction of metronidazole concentrations showed significant differences between the following concentrations: 5x300 mg/L, 50x300 mg/L at 24 h; 5x150 mg/L and 5x300 mg/L at 48 h; 5x150 mg/L and 5x300 mg/L at 72 h; 5x300 mg/L at 96 h. These results are similar to those of Carreira, et al.^1^ (2007) regarding the antimicrobial action of metronidazole, which found satisfactory results regarding the association with 4 µg/mL ciprofloxacin.

Results obtained using this methodology may serve as a motivation for new studies with the drugs used in this trial. It is important to include these findings in the critical analysis of the use of new drugs in intracanal dressing.

## CONCLUSION

Based on the obtained results, the following conclusions can be drawn: 1. All tested antibiotics (ciprofloxacin, clyndamicin and metronidazole) showed dose-dependent cytotoxicity; 2. Regardless of the antibiotic, cell viability at 24 h was greater than in the other experimental times; 3. Concentrations of 5 and 50 mg/L of all antibiotics produced viable fibroblasts at all experimental times.
